# Optimizing Rhizome Quality in *Ligusticum chuanxiong* Hort. Through High Maltose Concentration

**DOI:** 10.3390/plants14203125

**Published:** 2025-10-10

**Authors:** Hui-Yeong Jeong, Ho-Jun Son, Jun-Won Kang, Ji-Ah Kim

**Affiliations:** 1 Forest Medicinal Resources Research Center, National Institute of Forest Science, Yeongju-si 36040, Republic of Korea; hdud7281@korea.kr (H.-Y.J.); shj7400@korea.kr (H.-J.S.); 2 Department of Forestry, School of Forest Sciences and Landscape Architecture, Kyungpook National Univesity, Daegu 41566, Republic of Korea; jwkang15@knu.ac.kr

**Keywords:** acclimatization, carbohydrate, field growth, In Vitro

## Abstract

*Ligusticum chuanxiong* Hort. (*L. chuanxiong*) is a traditional medicinal food in East Asia. This study provides a comprehensive analysis of the effects of various carbohydrates on *L. chuanxiong*. It covers rhizome induction, acclimatization, and field growth. In the context of this study, the most effective carbohydrates for promoting rhizome induction in vitro to field growth ex vitro of *L. chuanxiong* were identified as maltose treatments with a concentration of 4% and 6%. It has been demonstrated that, particularly at a concentration of 4%, this treatment is particularly beneficial for in vitro rhizome development of *L. chuanxiong*. Following acclimatization, the 6% maltose treatments exhibited the highest fresh weight (3.3 ± 0.2 g). Following the process of field growth, there was a significant increase in the fresh weight of the rhizome under the 6% maltose treatment (160.8 ± 22.2 g) in comparison with the other treatments. This investigation is the first study on rhizome production of *L. chuanxiong*. It is clear that the appropriate carbohydrate treatment protocol is key to optimizing rhizome production and providing fundamental data for the best propagation of *L. chuanxiong*.

## 1. Introduction

The rhizome of *Ligusticum chuanxiong* Hort. (*L. chuanxiong*), belonging to the Apiaceae family, is traditionally used as a medicinal food in East Asia. Phthalides, such as Z-ligustilide, neocnidilide, 3-butyl-4-hydroxyphthalide, and senkyunolide A have been identified as its main bioactive compounds [1]. These compounds have been reported to exhibit various pharmacological effects, including anti-cerebral ischemia, anti-myocardial ischemia, blood vessel protection, anti-thrombotic, anti-hypertensive, anti-atherosclerosis, anti-spasmodic, anti-inflammatory, anti-cancer, anti-oxidant, and anti-asthma [2,3].

However, *L. chuanxiong* exhibits seed infertility [4], which is a major factor contributing to its reproductive disorders. Rhizome fragmentation is the primary method of propagation, but this approach carries a high risk of virus dissemination, negatively impacting yield and quality during cultivation. Therefore, in vitro propagation technology presents a viable alternative for the efficient production of *L. chuanxiong* rhizomes.

Micro rhizomes, which are produced through in vitro propagation, offer several advantages, including the induction of disease-free plants and germplasm conservation. Additionally, micro rhizomes can be stored for extended periods and easily transported. The induction of micro rhizomes has been reported in various bulbous plants, including *Zingiber officinale* [5], *Curcuma longa* [6], *Kaempferia galanga* [7], yam [8], and potato [9].

Under in vitro micropropagation, rhizomes receive nutrients from artificial media composed of macronutrients, vitamins, minerals, and plant growth regulators [10]. Carbohydrates, a crucial component of macronutrients, include monosaccharides (pentose and hexose), oligosaccharides, and polysaccharides (disaccharides and trisaccharide), classified based on their atomic arrangement [11]. Key carbohydrates utilized by plants include hexoses (mannose, glucose, fructose, and galactose), pentoses (arabinose, ribose, xylose), disaccharides (maltose, sucrose, lactose, cellobiose, trehalose), and trisaccharide (raffinose). Additionally, sugar alcohols such as sorbitol, mannitol, and glycerol serve as alternative carbohydrate sources [12]. Carbohydrates play a crucial role in promoting plant growth, enhancing nutrient availability, and maintaining suitable osmolarity, which influences cell division rates and morphogenesis [13]. Furthermore, carbohydrates are essential for rooting, acting as signaling molecules that interact with phytohormones and regulate various developmental processes [14].

For most plant species, sucrose has been widely used to promote rhizome formation. In ginger, sucrose concentrations between 60 and 90 g/L were most effective for rhizome induction [15]. Similarly, 50 g/L of sucrose successfully induced bamboo rhizomes [10]. However, sucrose is not always the most effective carbohydrate, as plants utilize various other sugars for growth and development [16]. In the Apiaceae family, limited research has examined plant growth responses to different carbohydrates. Among sucrose treatments of 30, 50, 70, and 90 g/L, 50 g/L was found to be optimal for rhizome induction in *Cnidium officinale* [17].

The promoting effects of high concentrations of maltose and sucrose on rhizome formation have also been reported in other plant species. For example, in potato, both sugars at 80 g/L were found to be equally effective in stimulating tuberization [18]. Likewise, in cocoyam, the greatest mini-tuber fresh weight and height were obtained with 80 g/L of maltose and 60 g/L of sucrose [19]. In *Ducrosia flabellifolia*, the highest shoot initiation was observed in a medium containing 30 g/L of D-sorbitol; however, none of the D-sorbitol treatments (0, 10, 20, 30, or 40 g/L) resulted in successful rooting [20]. The effects of sucrose, fructose, glucose, and maltose on shoot regeneration in *Centella asiatica* were also investigated, and the maximum shoot length was obtained in a medium supplemented with 40 g/L of sucrose [21].

Identifying the appropriate carbohydrate treatment for *L. chuanxiong* rhizome production requires evaluating various carbohydrate sources and their effects across both in vitro and ex vitro stages. Despite the recognized importance of carbohydrates in plant development, no study has systematically analyzed the impact of different carbohydrate types and concentrations on rhizome formation in *L. chuanxiong*. Moreover, research on *L. chuanxiong* tissue culture remains scarce. Therefore, in this study, we examined the effects of different carbohydrates from in vitro growth to field performance to determine their practical relevance. The main objectives were to identify the most suitable carbohydrate type and concentration for rhizome induction and to enhance the quality of *L. chuanxiong* rhizomes.

## 2. Results

### 2.1. Carbohydrate Treatment In Vitro

The effects of different carbohydrate treatments on in vitro growth characteristics were investigated. Plant height was significantly influenced by carbohydrate type. The greatest shoot length was observed in the 2% maltose (4.64 ± 0.69 cm) and 2% glucose (4.26 ± 0.45 cm) treatments (Table 1). In addition, the 2% maltose (8.07 ± 1.63 g) and 4% maltose (8.52 ± 2.47 g) treatments produced the longest roots. The highest number of roots was recorded in the 4% maltose treatment (10.92 ± 1.24), followed by 2% maltose (8.28 ± 1.58). Therefore, the 4% maltose treatment was the most effective in promoting root formation, followed by 2% maltose.

**Figure 1:**
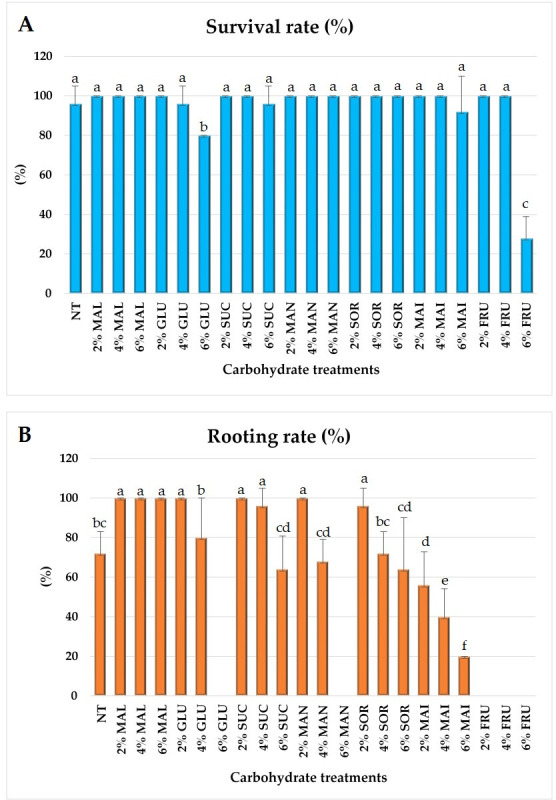
Survival rate (**A**) and rooting rate (**B**) of *L. chuanxiong* in vitro under different carbohydrate treatments. Control (NT, no treatment); maltose (MAL); glucose (GLU); sucrose (SUC); mannose (MAN); sorbitol (SOR); mannitol (MAI); fructose (FRU)). Means with the same letters are not significantly different by Duncan’s Multiple Range Test (DMRT, *p* < 0.05). Data are presented as means ± SD of five biological replicates, each consisting of five plants (*n* = 5).

**Figure 2:**
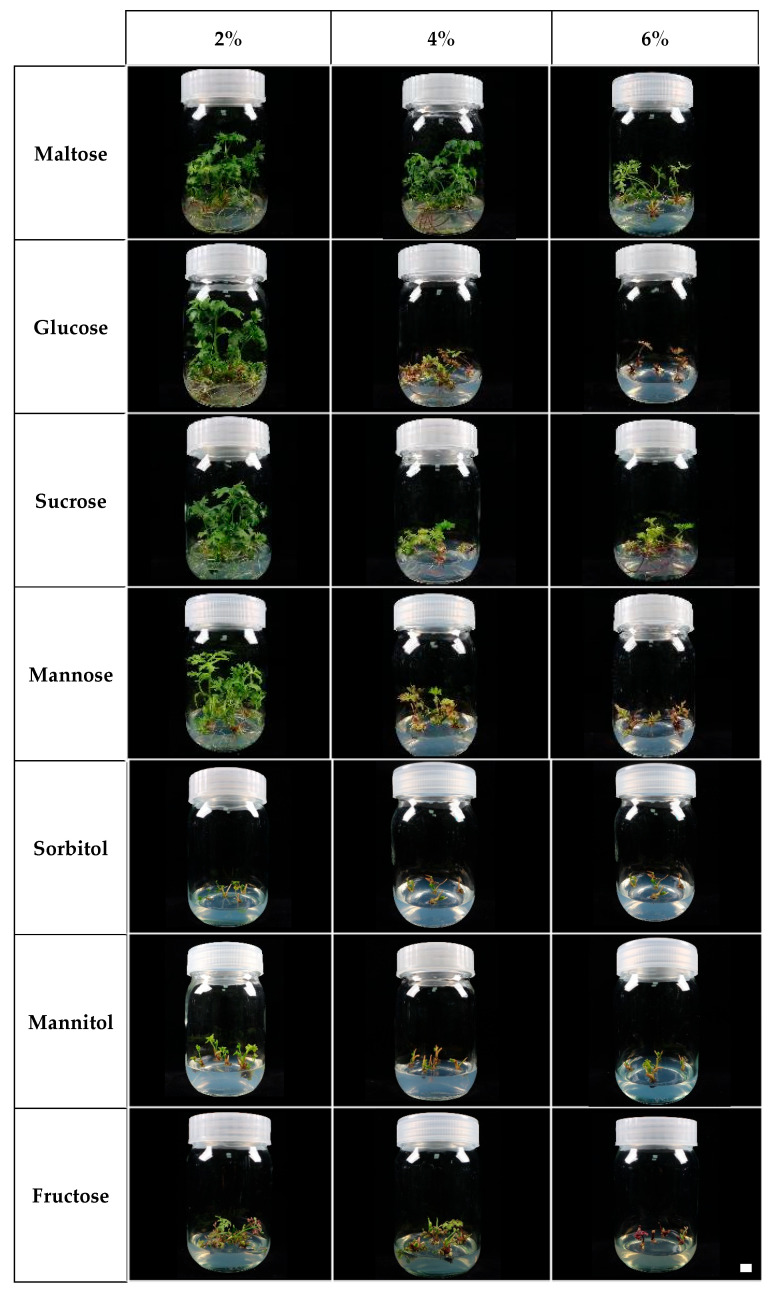
Effects of carbohydrate types and concentrations on rhizome formation in *L. chuanxiong* in vitro (scale bar = 1 cm).

Under in vitro carbohydrate treatments, all plantlets in the 2% concentration groups exhibited a 100% survival rate (Figure 1A). However, at higher concentrations, survival declined compared with the 2% treatments. Notably, 6% fructose (28%) and 6% glucose (80%) showed lower survival rates than the control (96%). Rooting success also varied among treatments. All maltose concentrations (2–6%) and the 2% glucose, sucrose, and mannose treatments achieved a 100% rooting rate (Figure 1B). In contrast, 6% glucose, 6% mannitol, and all fructose treatments (2–6%) completely inhibited rhizome formation. The overall morphological appearance of in vitro plantlets under different carbohydrate treatments is shown in Figure 2. Based on survival rates and rhizome formation, all plantlets were washed and transferred to pots for acclimatization to evaluate the ex vitro effects of carbohydrate treatment type and concentration.

### 2.2. Acclimatization and Investigation

**Figure 3:**
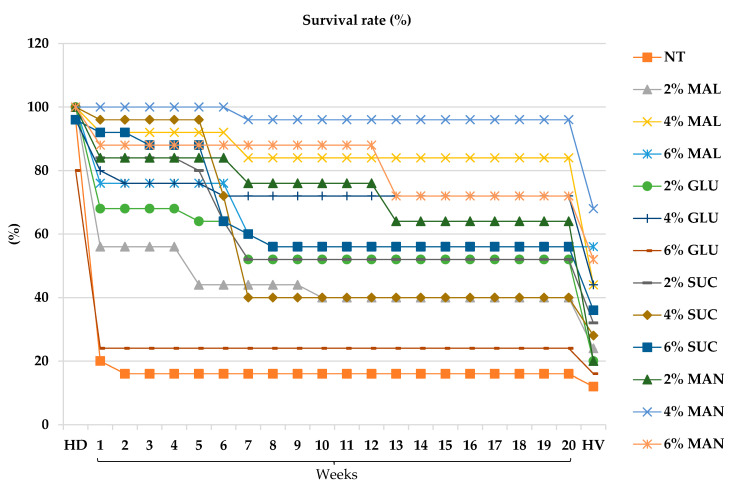
Survival rate of *L. chuanxiong* from hardening (HD) to harvest (HV) as affected by carbohydrate types and concentrations (*n* = 25). Control (NT, no treatment); maltose (MAL); glucose (GLU); sucrose (SUC); mannose (MAN). Numbers 1–20 indicate 7-day intervals.

Following acclimatization, plant growth characteristics, including survival rate, plant height, and number of leaves, were assessed weekly. The survival rate exhibited three distinct phases of decline (Figure 3). The first occurred between the hardening phase and the first week of observation, likely reflecting stress associated with the transition from in vitro to ex vitro conditions. The second decline was observed at the sixth week, indicating a temporary reduction in survival. The third decline occurred between the 20th week and harvest, possibly resulting from nutrient depletion or physiological aging.

The presence of carbohydrates positively influenced acclimatization success, with higher carbon source concentrations generally associated with increased survival rates. Among treatments, 4% mannose produced the highest survival rate after harvest (96%), followed by 4% maltose (84%). In contrast, all mannitol treatments (2%, 4%, and 6%) and the 2% sorbitol treatment resulted in complete plant mortality during acclimatization (Figure S1). The remaining sorbitol treatments (4% and 6%) exhibited extremely low survival rates (<8%).

At the 20th week, the tallest plants were recorded in the 4% mannose (9.8 cm), 4% sorbitol (9.6 cm), and 6% maltose (9.3 cm) treatments (Figure S2). Similarly, the highest number of leaves was observed in the 6% fructose (9.5 leaves) and 2% maltose (8.3 leaves) treatments (Figure S3). These results suggest that maltose and mannose enhance plant growth, whereas high concentrations of sorbitol and mannitol exert detrimental effects on plant survival and development.

### 2.3. First Harvest and Investigation

The rhizomes were harvested, and key growth parameters—including rhizome formation rate (survival rate), rhizome length, rhizome diameter, and fresh weight—were recorded (Table 2). Taken together, carbohydrate treatments improved the survival rate after acclimatization compared with the untreated control.

**Figure 4:**
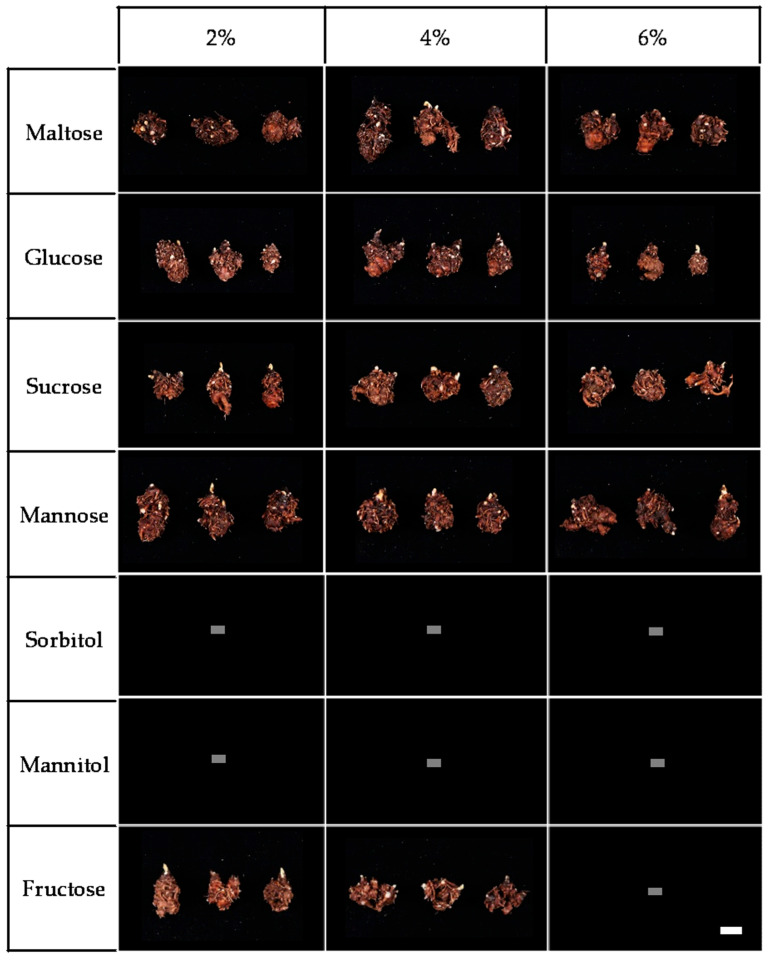
*L. chuanxiong* rhizome growth by carbohydrate types and concentrations after first harvest (scale bar= 1 cm).

**Figure 5:**
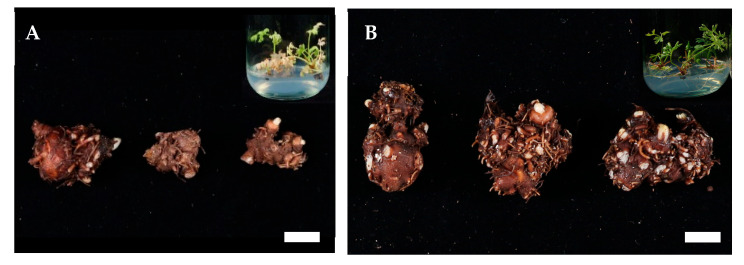
Rhizome formation of *L. chuanxiong* after the first harvest under control (**A**) and 6% maltose (**B**) treatments (scale bar = 1 cm). Inset shows the rhizome appearance from previous in vitro carbohydrate treatments.

The highest rhizome length was observed in the 2% mannose treatment. In addition, rhizome fresh weight showed outstanding results in both the 6% maltose (3.3 ± 0.2 g) and 2% mannose treatments (3.2 ± 0.3 g); however, the survival rate under 2% mannose was relatively low (20%). Similarly, the 4% fructose treatment resulted in the greatest rhizome diameter, but its survival rate was also low (28%). In contrast, the 6% maltose treatment exhibited a 56% survival rate. These results suggest that 6% maltose plays a crucial role in enhancing both the quality and quantity of rhizome formation. The maltose and mannose treatments (2%, 4%, and 6%) produced significantly larger and healthier rhizomes than the other groups (Figure 4), emphasizing the potential of carbohydrate sources in promoting *L. chuanxiong* rhizome production. Figure 5 illustrates the differences in rhizome formation between the control and the 6% maltose treatment.

### 2.4. Field Growth and Second Harvest

**Figure 6:**
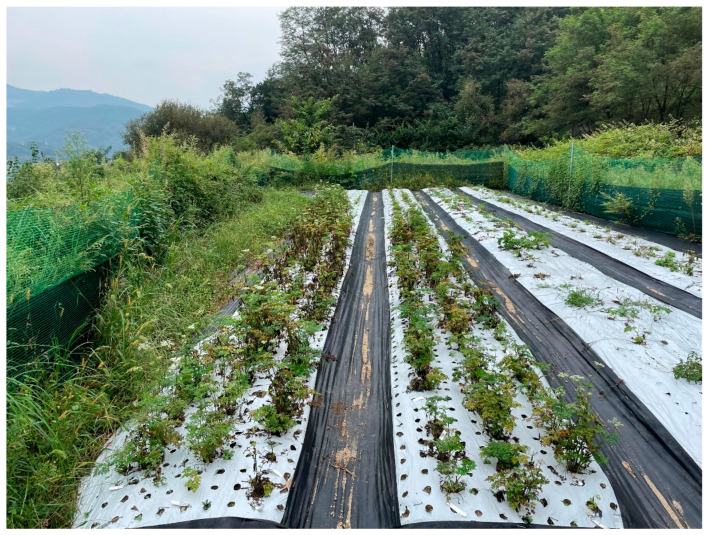
Photograph of field-grown *L. chuanxiong* in October.

After six months in the field (Figure 6), plant growth characteristics—including survival rate, plant height, and fresh weight—were assessed. Survival rates were 100% for most treatments, except for 4% and 6% mannose and 2% and 4% fructose, which showed slightly lower values (Table 3). Following the first harvest after acclimatization, survival rates varied among the carbohydrate treatments and the control; however, under field conditions, no significant differences were observed between treated and untreated plants. The tallest plants were observed in the 6% maltose treatment (37.4 ± 1.6 cm), followed by 4% maltose (30.6 ± 1.4 cm). In contrast, the smallest plants were recorded in the 2% sucrose treatment (18.7 ± 1.9 cm). Fresh weight was significantly higher under the 6% maltose treatment (160.8 ± 22.2 g) compared with the other treatments.

**Figure 7:**
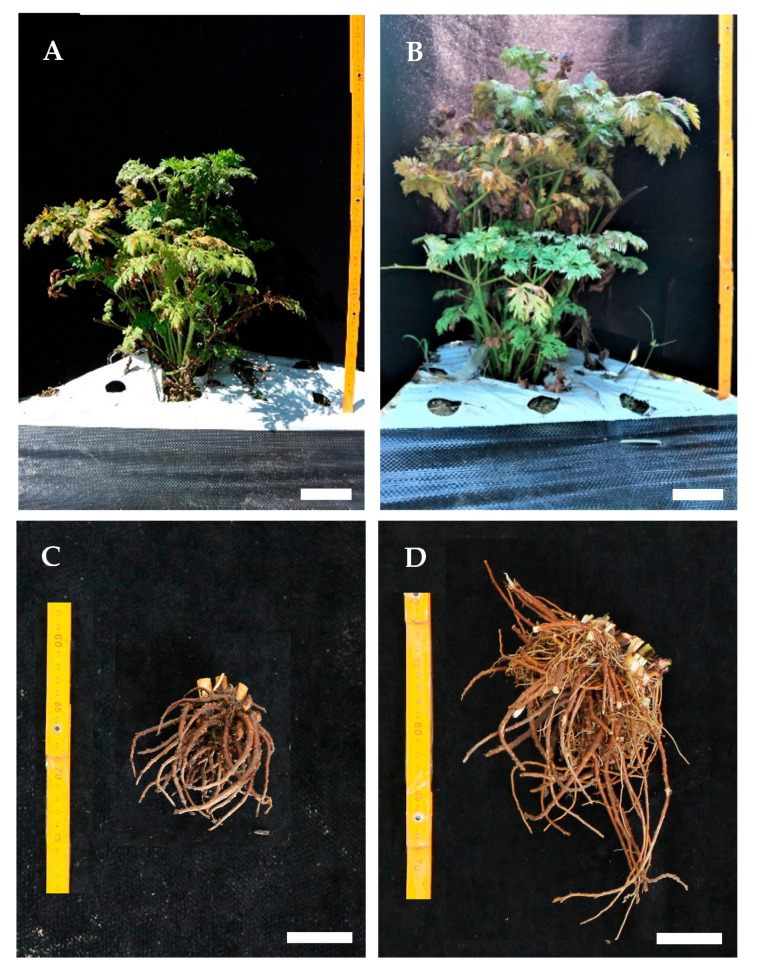
Photography of field growth and harvested rhizome of *L. chuanxiong* (**A**,**C**; control, **B**,**D**; 6% maltose) (scale bar = 5 cm).

The field growth and the harvested rhizome formation of control and 6% maltose treatment are shown in Figure 7. Compared with the control, the 6% maltose treatment was the most effective in promoting plant growth and rhizome formation (Figure 7).

**Figure 8:**
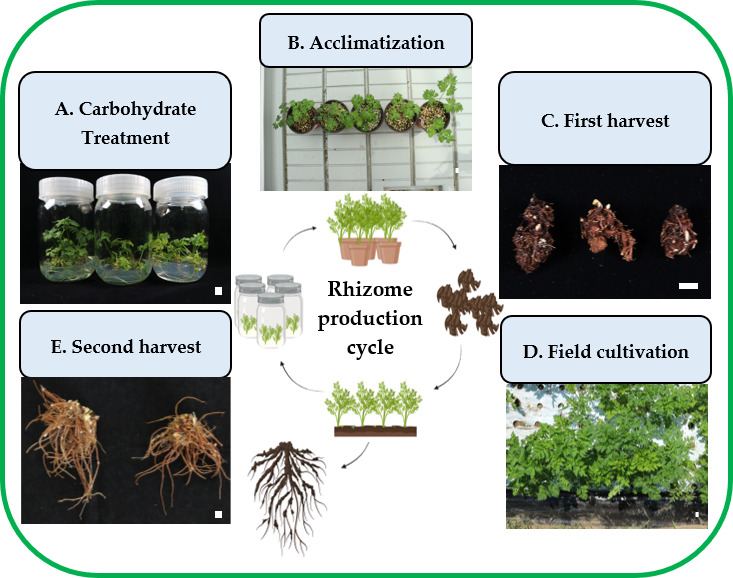
Process of rhizome production cycle of *L. chuanxiong* (carbohydrate treatment in vitro (**A**); acclimatization and plant growth (**B**); first harvest (**C**); field cultivation (**D**) and second harvest (**E**)) (scale bar = 1 cm). Illustrative figures are created with icon elements from BioRender (Biorender.com).

In general, a high concentration (≥4%) of maltose was found to be the superior treatment in promoting plant growth and rhizome formation among all experimental carbohydrate source treatments. The rhizome production process (Figure 8) in this study involved five key stages: carbohydrate treatment in vitro to optimize nutrient availability for initial plantlet development (Figure 8A), acclimatization in a smart greenhouse to enhance adaptation to external conditions (Figure 8B), rhizome harvesting to assess rhizome formation and biomass accumulation (Figure 8C), and field growth evaluation to determine long-term growth potential under natural conditions (Figure 8D), and enlarged rhizomes were harvested (Figure 8E).

## 3. Discussion

Carbohydrates not only serve as structural and nutritional components but also play more complex regulatory roles in plant growth and development [22,23]. A recent paper reported the carbon supply–consumption balance in plant roots that bridges root anatomical structures and root function [24]. It is also utilized as a technique to increase the success rate of acclimatization, which is a major step in tissue culture [5].

It remains unclear whether carbohydrates play a key regulatory role in the rhizome formation of *L. chuanxiong*. In this study, we investigated the function of carbon source and osmotic regulator, understanding its role in rhizome formation and enhancing the reproductive coefficient and efficiency of *L. chuanxiong.* In several carbohydrate treatments, though sucrose is the main organic carbon for most plants, 4% and 6% maltose yielded the outstanding results, indicating that elevated maltose levels enhance rhizome development in *L. chuanxiong*. This result suggests that maltose is an optimal carbohydrate source for *L. chuanxiong*, particularly at higher concentrations. The effectiveness of high concentrations of maltose and sucrose in promoting rhizome production has also been observed in other plant species. For instance, studies on potato tuberization demonstrated that both maltose and sucrose at 80 g/L were equally effective in inducing tuber formation [18]. Similarly, in cocoyam, the highest fresh weight and height of mini-tubers were achieved with 80 g/L of maltose and 60 g/L of sucrose [19]. Studies have shown that a high concentration of the carbon source can significantly promote the formation of tubers in vitro. The effectiveness of high carbohydrate concentrations in promoting tuberization may be attributed to their role in upregulating genes involved in nitrate assimilation, growth, storage, and starch remobilization [25]. Except for the 4% maltose treatment, all carbohydrate treatments above 4% inhibited rhizome growth and development in vitro, causing leaf wilting and stem atrophy in our study. This may be attributed to the osmotic stress induced by high sucrose concentrations [26].

In our study, carbohydrates exerted a positive influence on the acclimatization stage, with increasing concentrations of carbon sources generally enhancing the success rate of acclimatization. This finding indicates that the supplementation of exogenous carbohydrates provides an essential energy supply that facilitates the metabolic adjustment of plantlets when transitioning from in vitro conditions to ex vitro environments. Consistent with our results, previous studies have reported that carbohydrate treatments improve acclimatization efficiency by promoting osmotic balance, enhancing photosynthetic competence, and mitigating transplant stress [5]. Thus, the controlled application of carbohydrate sources represents an effective strategy to improve survival and growth during the acclimatization phase in plant tissue culture systems.

After careful consideration, 4% maltose was selected as the optimal carbohydrate concentration for inducing in vitro rhizome formation of *L. chuanxiong*.

In plants, maltose is mainly produced in chloroplasts, and MEX1 is a protein that is responsible for transferring it to the cytoplasm. MEX1 transports maltose from the chloroplasts to the cytoplasm, during which it can travel to the roots [27]. In addition, maltose may affect gene expression associated with sugar transport in the roots. For example, maltose may regulate sugar transport in the roots by regulating the expression of sugar transporters such as SWEET2 [28]. This report suggested that maltose may play an important role in regulating sugar metabolism and transport in the roots. Several carbohydrate metabolism genes have been identified, including Sucrose Synthase 1 (*SUS1*), Cell Wall Invertase 1 (*CWINV1*), Hexokinase 1 (*HXK1*), Fructokinase-like 1 (*FLN1*), Starch Synthase 3 (*SS3*), and Sucrose-Proton Symporter 5 (*SUC5*) [29].

Our results report that maltose is not only a metabolic product but also influences rhizome development. Based on the second harvest results, it is evident that 6% maltose treatment provided the most favorable conditions for plant growth and rhizome formation among all tested carbon sources. The 6% maltose treatment exhibited the greatest height and a pronounced stimulation of rhizome development compared to the control and other treatments. This enhanced growth performance indicates that maltose at an optimal concentration may serve not only as a readily available energy source but also as a regulatory factor influencing carbon allocation toward storage organ development. The significant increase in fresh weight after harvest further supports the role of maltose in promoting biomass accumulation, suggesting that its application could be a valuable strategy for improving yield and enhancing underground storage organ formation in plants. However, under in vitro culture conditions, 6% maltose treatment showed reduced growth performance; instead, 4% maltose was concluded to be more suitable for the production of rhizomes. Overall, a high concentration (≥4%) of maltose treatment was effective for rhizome formation of *L. chuanxiong* from in vitro to ex vitro plant growth and development.

In addition, maltose has a positive effect on rhizome development when incubated with other carbohydrates. For example, maltose and trehalose have been shown to increase the growth and yield of wheat under dry stress [30]. Also, it suggested that maltose can promote rhizome development of plants even under stress conditions. This report suggested that maltose may play an important role in the rhizome development of plants, and further studies on the function and mechanism of maltose are needed in the future.

In contrast, mannitol treatment produced the poorest results, reinforcing previous findings that mannitol is ineffective for rhizome development. Studies have reported complete inhibition of rooting in both gladiolus [31] and apple rootstocks [32] when grown in media containing mannitol. The inability of certain plants to utilize mannitol as a carbohydrate source is likely due to the absence of NAD-dependent mannitol 1-oxidoreductase (MDH), which is an enzyme essential for mannitol metabolism in sink tissues [33]. Mannitol and sorbitol treatments mainly decreased the osmotic potential through the uptake, transport, and accumulation of mannitol and sorbitol treatments, respectively, and to a certain extent, through that of maltose, leading to the most intense decrease in the relative water content, chlorophyll, and photosynthetic activity of the wheat leaves [34]. However, physiological responses to mannitol are not always similar. Mannitol at 2% increased the root number and photosynthetic pigment levels, along with PEG pretreatment, in the in vitro tuberization of *Dactylorhiza umbrosa* [35].

This study showed that maltose supplementation promoted superior plant growth compared with sucrose, which is usually used as the main carbon source. These results support earlier findings that maltose reduces callus browning and enhances regeneration efficiency [36]. Maltose is rapidly hydrolyzed into glucose by maltase, providing an immediate energy source, whereas sucrose requires invertase activity, making its metabolic pathway more complex [24]. Such differences likely contribute to the improved growth observed in maltose-containing media.

In the present study, among the maltose treatments, 4% maltose was the most effective for in vitro growth, whereas 6% maltose showed better performance during ex vitro acclimatization. These results are consistent with previous reports, indicating that high concentrations of carbon sources may reduce in vitro growth due to osmotic stress or metabolic imbalances, but can enhance ex vitro survival and growth by supporting the accumulation of reserve carbohydrates and improving stress tolerance [37]. These findings emphasize the importance of selecting both the carbohydrate type and concentration according to species and developmental stage to improve growth and survival.

In general, sucrose is used as the main carbon source, so we compared the actual cost and practicality with maltose, which was found to be more effective in this study. Based on the reagent prices used in this experiment, the raw material cost of maltose is approximately eight times higher than that of sucrose, but the yield is more than four times higher. Although simple cost/output ratios may appear uneconomical, high productivity, stability under maltose conditions, potential for cost reduction in downstream processes, and the good quality of the final product can justify the additional costs. This indicates that the use of maltose can be justified from both an industrial and practical perspective.

This study clearly demonstrates that *L. chuanxiong* requires a high concentration (≥4%) of maltose for optimal rhizome production as observed across the rhizome induction, acclimatization, and field stage. However, rhizome formation is influenced by multiple factors beyond carbohydrate type and concentration. Further studies should consider additional physiological and environmental variables to refine the optimization process for rhizome production of *L. chuanxiong*.

## 4. Materials and Methods

### 4.1. Plant Material

The rhizomes of *L. chuanxiong* were collected in Bonghwa-gun, Gyeongsangbuk-do, Republic of Korea. Samples were washed under running tap water to remove soil, disinfected in 70% ethanol (EtOH) for 2 min, and washed three times with sterile distilled water. Subsequently, they were treated with 4% NaOCl solution for 30 min and washed five times with sterile distilled water. Buds were excised from the rhizomes and decontaminated on MS medium (Duchefa Biochemie, Haarlem, The Netherlands), supplemented with 100 mg/L of tetracycline. Experimental materials were obtained from in vitro plants derived from buds subcultured more than ten times on half-strength MS medium supplemented with 2% sucrose and 0.3% gelrite (pH 5.7) at monthly intervals.

### 4.2. Carbohydrate Treatment

Shoots were cut to approximately 1.5–2.0 cm in length, and roots were completely removed. The control treatment consisted of MS medium containing full-strength MS salts, 2% sucrose, and 0.3% gelrite (pH 5.7). In addition, seven carbohydrate sources (Duchefa Biochemie, Haarlem, The Netherlands)—maltose, glucose, sucrose, mannose, sorbitol, mannitol, and fructose—were tested at concentrations of 2%, 4%, and 6%.

### 4.3. In Vitro Culture Conditions and Data Collection

All media were sterilized in an autoclave (196 L, HS-50200, Hanshin Medical Corporation, Incheon, Republic of Korea) at 121 °C for 15 min. Glass bottles (H: 13.0 cm × W: 7.7 cm; SPL Life Science Inc., Pocheon, Republic of Korea) were used as culture vessels. The bottles were kept in a growth room controlled by an environmental system (Korea Science and Technology Industry, Suwon, Republic of Korea) at 25 ± 1 °C and 45 ± 2% relative humidity. Cultures were maintained under a 16 h photoperiod with photosynthetically active radiation (70 μmol·m^−2^·s^−1^) provided by fluorescent lamps (FHF32W/865, OSRAM, Munich, Germany). Before acclimatization, plant growth characteristics, including survival rate, plant height, number of leaves, rooting rate, root length, and number of roots, were recorded. Root length was measured as the length of the longest root.

### 4.4. Acclimatization and Field Growth

Twenty-five in vitro plants from each treatment were washed and transferred to containers (H: 10.6 cm × W: 11.5 cm; GS120, Joy Garden, Icheon, Republic of Korea). Three types of artificial soil—peat moss (Shinsung Mineral, Goesan, Republic of Korea), perlite, and vermiculite (Green Fire Chemicals, Hongseong, Republic of Korea)—were mixed in a 1:1:1 ratio. Residual medium was removed from the rhizomes using tap water before acclimatization. During the first two weeks, humidity was maintained at 90%, followed by a reduction to 60% for the next two weeks. The acclimatization room was maintained at 25 ± 1 °C with a 16 h photoperiod under photosynthetically active radiation (70 μmol·m^−2^·s^−1^) provided by white fluorescent lamps. After one month, plants were transferred to a smart greenhouse. Growth parameters, including survival rate, plant height, and number of leaves, were recorded every 7 days. After 12 months of acclimatization, rhizomes were harvested, and rootlets were removed. The following parameters were measured: rhizome formation rate, rhizome width, rhizome length, and rhizome fresh weight. A total of 131 harvested rhizomes were planted in the field in April at 36°50′00″ N, 128°31′35″ E, at an elevation of 373 m above sea level. After four months, plant growth characteristics, including plant height and survival rate, were recorded. Additionally, photographs were taken in August to compare plant growth based on different carbohydrate types and concentrations. Final harvesting was carried out in October, after which the rhizomes were photographed and their fresh weight recorded.

### 4.5. Statistical Analysis

To optimize carbohydrate conditions, the study was conducted in three phases: rhizome induction in vitro, acclimatization, and field growth. For in vitro culture, each treatment consisted of five biological replicates, each containing five independent plantlets. Following acclimatization, surviving plantlets were transferred and harvested after twelve months. The length, width, and fresh weight of rhizomes were assessed as growth parameters using three biological replicates. Subsequently, surviving plants were transplanted to the field and harvested after six months. Plant height and fresh weight were assessed as growth parameters using three biological replicates. Statistical analysis was performed using SPSS software (IBM SPSS Statistics, version 25; IBM Co., Armonk, NY, USA). Data were analyzed using analysis of variance (ANOVA), followed by Duncan’s Multiple Range Test (DMRT) at a significance level of *p* < 0.05.

## 5. Conclusions

This study provides a comprehensive analysis of the effects of various carbohydrates on *L. chuanxiong* rhizome induction, acclimatization, and field growth. The results highlight maltose, particularly at higher concentrations (4% and 6%), as the most effective carbohydrate for promoting rhizome formation and plant growth. Mannose also showed promising effects on rhizome size but was less effective in increasing the overall rhizome formation rate. Conversely, sorbitol and mannitol had detrimental effects, leading to low survival rates and poor growth. These findings suggest that carbohydrate selection plays a crucial role in optimizing rhizome production and field performance, providing valuable insights for large-scale propagation of *L. chuanxiong*. Further research should explore the underlying mechanisms of carbohydrate metabolism and its impact on rhizome development at the molecular level.

## Figures and Tables

**Table 1:** Impacts of carbohydrate types and concentrations on *L. chuanxiong* rhizome production in vitro culture.

Carbohydrate Type	Carbohydrate Conc. (%)	Shoot Length (cm)	No. of Roots/Explant	Root Length (cm)
Control	-	2.44 ± 0.49 def	1.96 ± 0.48 ghi	1.56 ± 0.47 hi
Maltose	2	4.64 ± 0.69 a	8.28 ± 1.58 b	8.07 ± 1.63 a
	4	3.08 ± 0.62 bc	10.92 ± 1.24 a	8.52 ± 2.47 a
	6	2.15 ± 0.39 efgh	5.48 ± 0.81 f	4.75 ± 1.05 cd
Glucose	2	4.26 ± 0.45 a	7.16 ± 0.82 cd	6.90 ± 0.82 b
	4	1.98 ± 0.26 fghij	2.24 ± 0.36 gh	2.28 ± 0.66 gh
	6	1.36 ± 0.10 klm	-	-
Sucrose	2	2.86 ± 0.63 cd	7.60 ± 0.58 bc	5.25 ± 0.46 c
	4	1.64 ± 0.22 hijk	5.68 ± 0.70 ef	3.75 ± 1.23 def
	6	1.67 ± 0.41 ghijk	2.00 ± 0.60 ghi	3.39 ± 0.57 ef
Mannose	2	3.50 ± 0.51 b	6.44 ± 0.82 de	4.41 ± 1.04 cde
	4	2.06 ± 0.29 efghi	2.32 ± 0.63 gh	1.46 ± 0.29 hi
	6	1.31 ± 0.13 klm	-	-
Sorbitol	2	2.18 ± 0.45 efg	2.84 ± 0.67 g	3.20 ± 0.51 fg
	4	1.17 ± 0.19 klm	1.28 ± 0.39 ijk	1.44 ± 0.29 hi
	6	1.13 ± 0.09 klm	1.04 ± 0.68 jkl	1.00 ± 0.53 ij
Mannitol	2	1.49 ± 0.20 jkl	1.80 ± 0.42 hij	0.84 ± 0.39 ij
	4	1.02 ± 0.08 lm	0.60 ± 0.32 klm	0.26 ± 0.12 j
	6	0.92 ± 0.17 mn	0.24 ± 0.09 lm	0.10 ± 0.02 j
Fructose	2	2.54 ± 0.46 de	-	-
	4	1.53 ± 0.21 ijkl	-	-
	6	0.47 ± 0.10 n	-	-

Means with the same letters are not significantly different by Duncan’s Multiple Range Test (DMRT, *p* < 0.05). Data are presented as means ± SD of five biological replicates, each consisting of five plants (*n* = 5).

**Table 2:** Impacts of carbohydrate types and concentrations on *L. chuanxiong* rhizome production after acclimatization.

Carbohydrate		Survival Rate (%)	Rhizome Characteristics		
Type	Conc. (%)		Length (cm)	Diameter (cm)	Fresh Weight (g)
Control	-	12	1.8 ± 0.5 cde	1.4 ± 0.2 abc	0.7 ± 0.1 f
Maltose	2	24	1.9 ± 0.1 cde	1.9 ± 0.5 abc	2.3 ± 0.4 bcd
	4	44	2.8 ± 0.8 ab	1.7 ± 0.3 abc	2.6 ± 0.6 b
	6	56	2.0 ± 0.3 bcde	1.9 ± 0.4 ab	3.3 ± 0.2 a
Glucose	2	24	1.3 ± 0.2 e	1.1 ± 0.1 c	0.6 ± 0.1 f
	4	44	1.6 ± 0.4 de	1.6 ± 0.8 abc	1.1 ± 0.5 ef
	6	16	1.8 ± 0.2 cde	1.4 ± 0.2 abc	1.0 ± 0.2 f
Sucrose	2	32	2.2 ± 0.4 bcd	1.5 ± 0.6 abc	1.7 ± 0.2 de
	4	28	2.5 ± 0.4 abc	1.3 ± 0.4 bc	1.9 ± 0.3 cd
	6	36	2.1 ± 0.2 bcd	2.0 ± 0.4 ab	1.7 ± 0.1 de
Mannose	2	20	3.0 ± 0.3 a	1.8 ± 0.2 abc	3.2 ± 0.3 a
	4	68	2.0 ± 0.2 cde	1.6 ± 0.2 abc	2.4 ± 0.3 bc
	6	52	2.5 ± 0.5 abc	1.8 ± 0.3 abc	2.1 ± 0.3 bcd
Sorbitol	2	-	-	-	-
	4	-	-	-	-
	6	-	-	-	-
Mannitol	2	-	-	-	-
	4	-	-	-	-
	6	-	-	-	-
Fructose	2	36	2.5 ± 0.3 abc	1.6 ± 0.2 abc	1.9 ± 0.3 cd
	4	28	2.1 ± 0.3 bcde	2.1 ± 0.5 a	1.9 ± 0.6 cd
	6	-	-	-	-

Means with the same letters are not significantly different by Duncan’s Multiple Range Test (DMRT, *p* < 0.05). Data are presented as mean ± SD, *n* = 3.

**Table 3:** Effects of carbohydrate types and concentrations on *L. chuanxiong* field growth.

Type	Conc. (%)	Survival Rate (%)	Plant Height	Fresh Weight (g)
Control	-	100	28.7 ± 7.5 bc	41.0 ± 15.2 cd
Maltose	2	100	29.8 ± 0.9 bc	88.5 ± 17.4 b
	4	100	30.6 ± 1.4 b	61.7 ± 4.1 c
	6	100	37.4 ± 1.6 a	160.8 ± 22.2 a
Glucose	2	100	30.1 ± 0.8 bc	30.1 ± 5.5 d
	4	100	26.3 ± 1.4 bc	54.4 ± 24.7 cd
	6	100	29.9 ± 1.2 bc	42.1 ± 6.6 cd
Sucrose	2	100	18.7 ± 1.9 e	46.1 ± 1.0 cd
	4	100	23.5 ± 2.7 cde	49.8 ± 10.2 cd
	6	100	19.6 ± 2.7 de	62.6 ± 27.3 c
Mannose	2	100	25.1 ± 7.4 bcd	53.3 ± 7.0 cd
	4	89	27.6 ± 5.4 bc	58.6 ± 2.6 c
	6	85	25.4 ± 0.4 bcd	63.2 ± 15.3 c
Sorbitol	2	-	-	-
	4	-	-	-
	6	-	-	-
Mannitol	2	-	-	-
	4	-	-	-
	6	-	-	-
Fructose	2	89	26.6 ± 1.9 bc	45.5 ± 2.5 cd
	4	86	29.9 ± 3.1 bc	35.8 ± 12.9 cd
	6	-	-	-

Means with the same letters are not significantly different by Duncan’s Multiple Range Test (DMRT, *p* < 0.05). Data are presented as mean ± SD, *n* = 3.

## Data Availability

The original contributions presented in this study are included in the article/supplementary material. Further inquiries can be directed to the corresponding author.

## Supplementary Materials

The following supporting information can be downloaded at https://www.mdpi.com/article/10.3390/plants14203125/s1, Figure S1: Survival rate of L. chuanxiong under different carbohydrate types and concentrations (n = 25). Control (NT, no treatment); maltose (MAL); glucose (GLU); sucrose (SUC); mannose (MAN); sorbitol (SOR); mannitol (MAI); fructose (FRU)). Numbers 1–20 indicate 7-day intervals.; Figure S2: Plant height of L. chuanxiong under different carbohydrate types and concentrations (n = 25). Control (NT, no treatment); maltose (MAL); glucose (GLU); sucrose (SUC); mannose (MAN); sorbitol (SOR); mannitol (MAI); fructose (FRU)). Numbers 1–20 indicate 7-day intervals.; Figure S3: Number of leaves of L. chuanxiong under different carbohydrate types and concentrations (n = 25). Control (NT, no treatment); maltose (MAL); glucose (GLU); sucrose (SUC); mannose (MAN); sorbitol (SOR); mannitol (MAI); fructose (FRU)). Numbers 1–20 indicate 7-day intervals.
