# Supplementary material for: Optimizing Rhizome Quality in Ligusticum chuanxiong Hort. Through High Maltose Concentration
Source: Plants (Basel). 2025 Oct 10;14(20):3125. doi: 10.3390/plants14203125 (PMC12566882; doi:10.3390/plants14203125)
Supplement: Supplementary file 1 [file plants-14-03125-s001.zip › plants-3906558_supplementary materials.pdf]

Supplementary materials

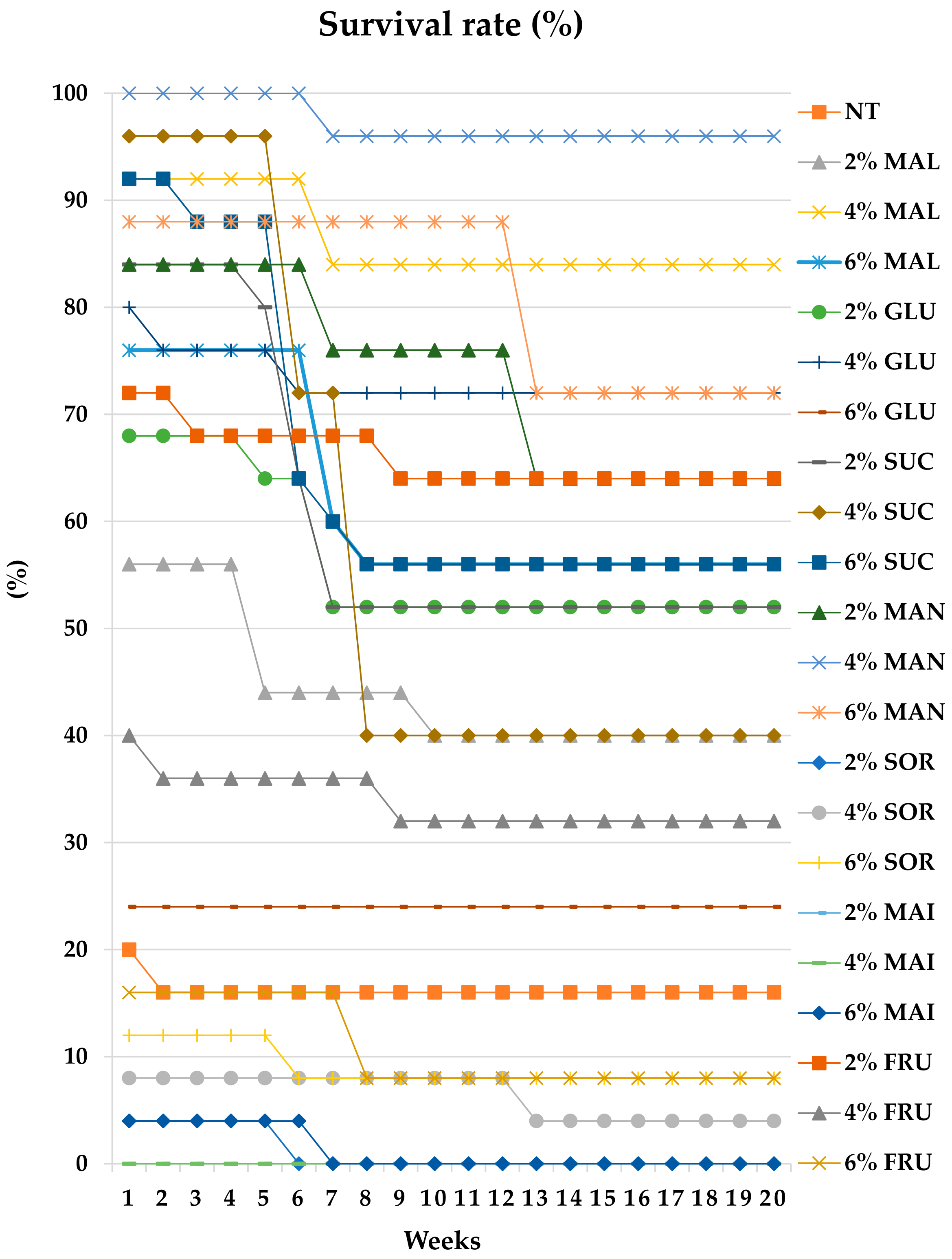

Figure S1. Survival rate of *L. chuanxiong* under different carbohydrate types and concentrations (*n* = 25). Control (NT, no treatment); maltose (MAL); glucose (GLU); sucrose (SUC); mannose (MAN); sorbitol (SOR); mannitol (MAI); fructose (FRU)). Numbers 1–20 indicate 7-day intervals.

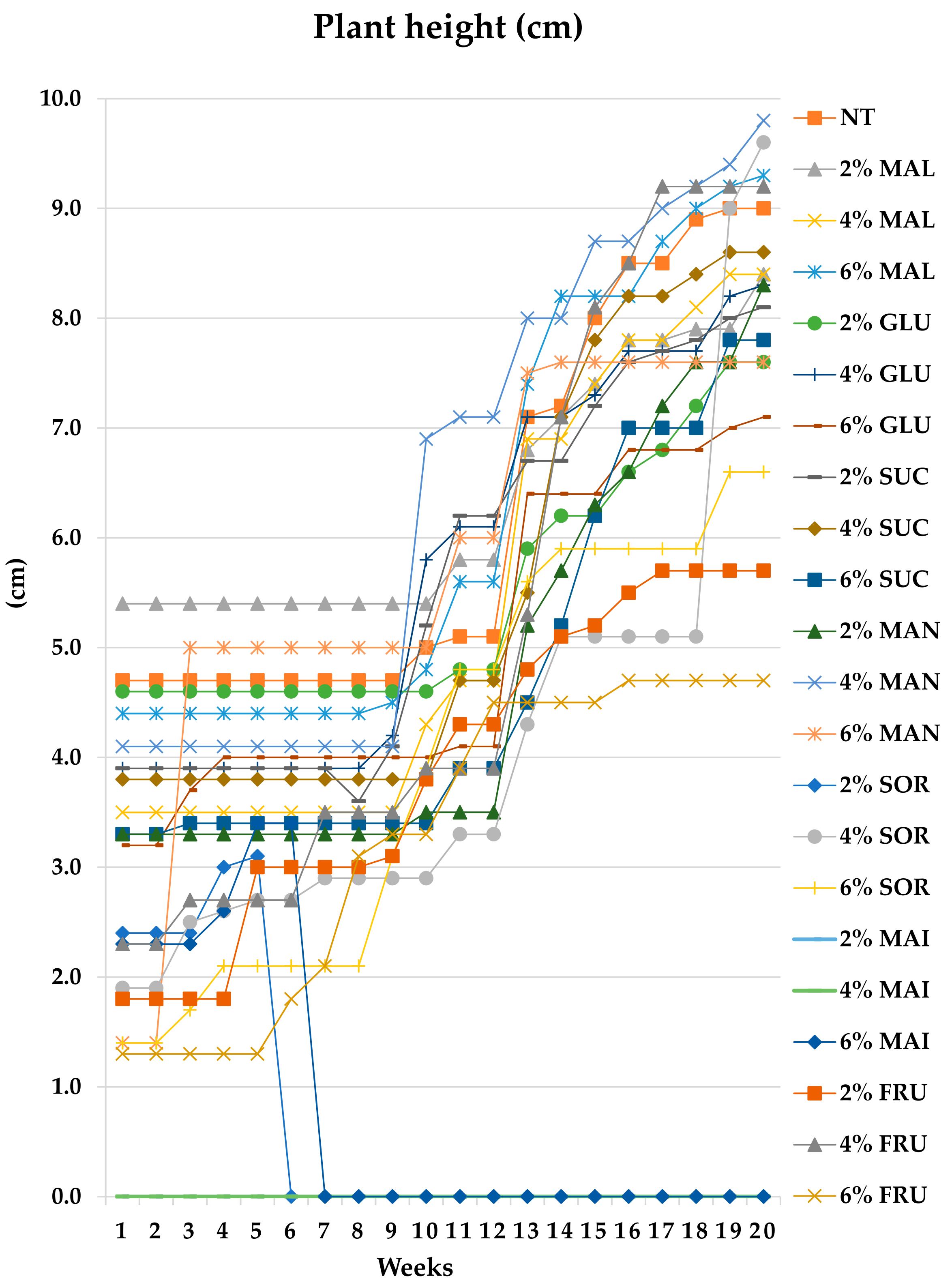

Figure S2. Plant height of *L. chuanxiong* under different carbohydrate types and concentrations (n = 25). Control (NT, no treatment); maltose (MAL); glucose (GLU); sucrose (SUC); mannose (MAN); sorbitol (SOR); mannitol (MAI); fructose (FRU)). Numbers 1–20 indicate 7-day intervals.

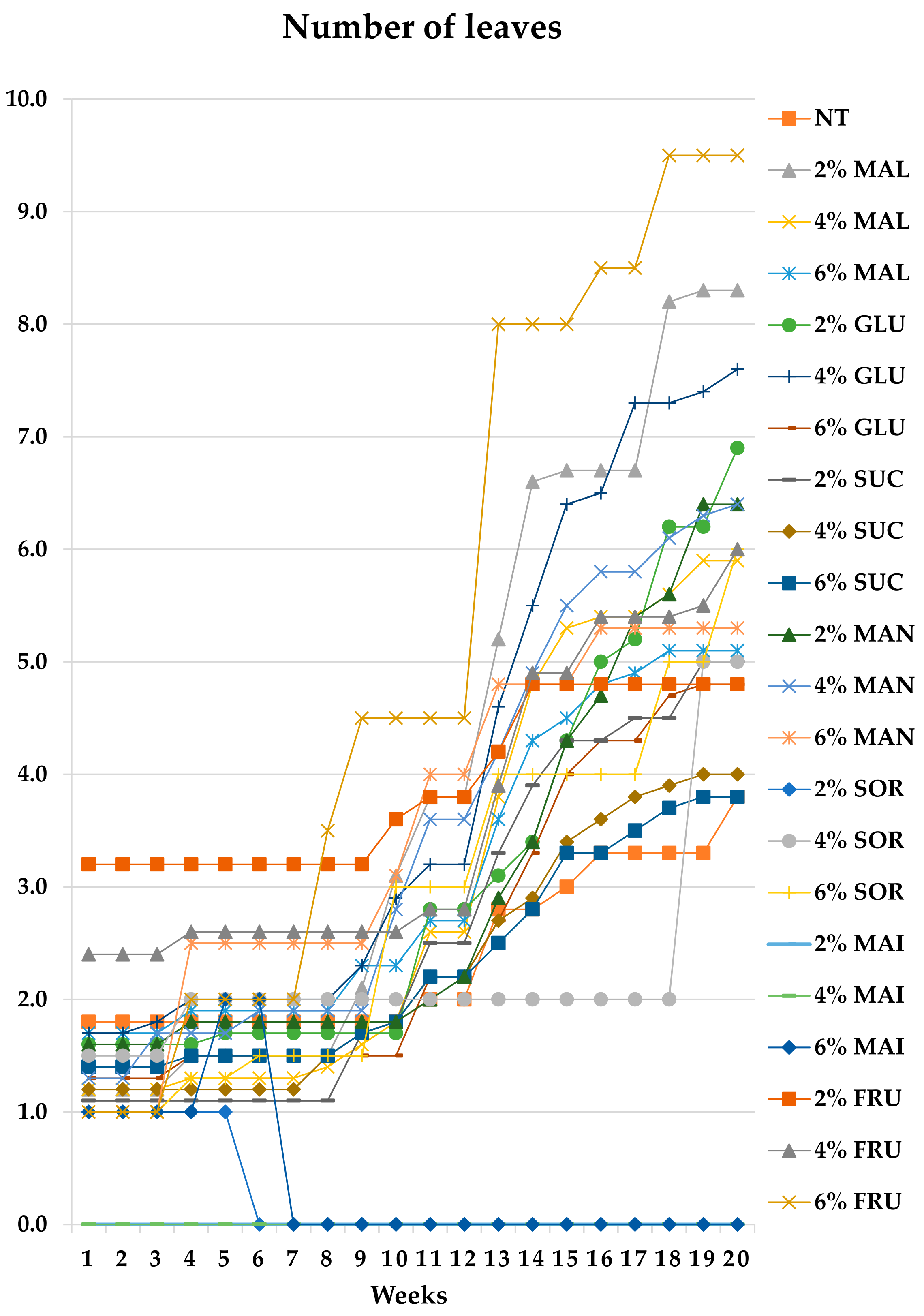

Figure S3. Number of leaves of *L. chuanxiong* under different carbohydrate types and concentrations (n = 25). Control (NT, no treatment); maltose (MAL); glucose (GLU); sucrose (SUC); mannose (MAN); sorbitol (SOR); mannitol (MAI); fructose (FRU)). Numbers 1–20 indicate 7-day intervals.
